# Structural basis for broad neutralization of ebolaviruses by an antibody targeting the glycoprotein fusion loop

**DOI:** 10.1038/s41467-018-06113-4

**Published:** 2018-09-26

**Authors:** Benjamin M. Janus, Nydia van Dyk, Xuelian Zhao, Katie A. Howell, Cinque Soto, M. Javad Aman, Yuxing Li, Thomas R. Fuerst, Gilad Ofek

**Affiliations:** 1 Institute for Bioscience and Biotechnology Research, University of Maryland, Rockville, MD 20850 USA; 2 Department of Cell Biology and Molecular Genetics, University of Maryland, College Park, MD 20742 USA; 3 Integrated BioTherapeutics, Rockville, MD 20850 USA; 4 The Vanderbilt Vaccine Center, Vanderbilt University Medical Center, Nashville, TN 37232 USA; 5 Department of Pediatrics, Vanderbilt University Medical Center, Nashville, TN 37232 USA; 6 Department of Microbiology and Immunology, University of Maryland School of Medicine, Baltimore, MD 21201 USA

## Abstract

The severity of the 2014–2016 ebolavirus outbreak in West Africa expedited clinical development of therapeutics and vaccines though the countermeasures on hand were largely monospecific and lacked efficacy against other ebolavirus species that previously emerged. Recent studies indicate that ebolavirus glycoprotein (GP) fusion loops are targets for cross-protective antibodies. Here we report the 3.72 Å resolution crystal structure of one such cross-protective antibody, CA45, bound to the ectodomain of Ebola virus (EBOV) GP. The CA45 epitope spans multiple faces of the fusion loop stem, across both GP1 and GP2 subunits, with ~68% of residues identical across > 99.5% of known ebolavirus isolates. Extensive antibody interactions within a pan-ebolavirus small-molecule inhibitor binding cavity on GP define this cavity as a novel site of immune vulnerability. The structure elucidates broad ebolavirus neutralization through a highly conserved epitope on GP and further enables rational design and development of broadly protective vaccines and therapeutics.

## Abstract (web summary)

The Ebola virus glycoprotein is a target for cross-protective antibodies. Here, Janus et al. report the crystal structure of the antigen-binding fragment of a pan-reactive antibody bound to a conserved epitope of the glycoprotein, facilitating rational design of cross-protective vaccines and therapeutics.

## Introduction

The genus ebolavirus of the family *filoviridae* is composed of five species, including Zaire (EBOV), Sudan (SUDV), Bundibugyo (BDBV), Reston (RESTV), and Taï-Forest (TAFV). Three of these species, EBOV, SUDV, and BDBV, have previously led to fatal outbreaks in humans. The 2014–2016 outbreak of EBOV strain Makona in West Africa, the most lethal outbreak documented thus far, resulted in over 28,000 cases and 11,000 deaths^1^. Its emergence in an unexpected geographical location and possible zoonotic adaptation to human cells indicates future outbreaks, including of other ebolavirus species, could emerge with unforeseen characteristics^2,3^. As such, development of vaccines and therapeutics that are cross-protective and focus on vulnerable regions of the virus that are conserved across ebolaviruses can offer potentially more durable protection less prone to escape or to the idiosyncrasies of any one emerging species. Several recent studies have reported isolation of cross-protective antibodies against ebolaviruses, including from human survivors and immunized animals, with some of the most broad and potent antibodies shown to target epitopes near or within the glycoprotein (GP) fusion loop at the GP1–GP2 subunit interface of GP^4–7^.

Fusion loops of membrane enveloped viruses form an integral component of the molecular machinery that mediates fusion of the virus and cell membranes during virus entry. In ebolaviruses, the fusion loop is encoded by N-terminal residues of the GP2 subunit and is composed of a hydrophobic internal fusion peptide flanked by an extended fusion loop stem, made up of GP2 strand β19–β20, and a disulfide-bonded base^8^. In the prefusion state of GP, which is the target conformation of neutralizing and protective antibodies, the fusion loop is found wrapped around the outer equatorial surface of the glycoprotein, forming a close interface with the GP1 core^8^. Engagement with the host-cell endosomal receptor Niemann-Pick C1-C (NPC1-C) and possible additional triggers are thought to lead to conformational rearrangement of the GP subunits, release of the fusion loop, insertion of the fusion peptide into the host-cell endosomal membrane, followed by formation of helical repeat hairpins that catalyze virus–host cell membrane fusion^9–13^. Various subregions of the ebolavirus fusion loop have been reported to be targeted by neutralizing antibodies, as also described for other viruses^14^,^15^, likely a result not only of the loop’s functional role but also, in the case of ebolaviruses, its placement along the outer GP1–GP2 interface of GP^8,16–18^.

Recently, a potent cross-protective antibody, CA45, was isolated from cynomolgus macaques immunized with trivalent cocktails of recombinant EBOV, SUDV, and Marburg virus (MARV) GPs. The CA45 antibody was shown by saturation mutagenesis and an 11 Å electron microscopic (EM) reconstruction to bind GP in the vicinity of the GP fusion loop^7^, with its binding site partially overlapping the binding sites of antibodies KZ52, c2G4, and c4G7^5,7^. In contrast to these latter antibodies, however, CA45 was found to be near pan-ebolavirus reactive with the capacity to recognize the GP ectodomains of four out of five ebolavirus species—EBOV, SUDV, BDBV, and RESTV—at binding affinities of 10.6, 3.3, 1.2, and 161 nM, respectively^7^. Low pH conditions were found to improve CA45 binding to GP, suggesting the antibody can maintain interaction with the virus even after trafficking into acidic endosomal compartments^7^. CA45’s breadth of GP recognition was reported to extend to its breadth of virus neutralization and protection. Depending on the viral assay and species, CA45 neutralized with IC_50_ values ranging from 0.9 to 78 nM, while it protected against EBOV, SUDV, and BDBV virus challenge in mice, guinea pigs, or ferrets, when administered alone (against SUDV) or in conjunction with a previously characterized receptor-binding region antibody FVM04 (against EBOV and BDBV)^5,7,19^.

To uncover the structural basis for the breadth of CA45-mediated ebolavirus neutralization, we determined the crystal structure of the fragment of antigen binding (Fab) of antibody CA45 bound to a mucin-domain deleted ectodomain of EBOV GP. The structure shows that CA45 targets the stem of the GP fusion loop and mediates extensive interactions with both its GP1 and GP2 subunits. Dissection of sequence variation within the CA45 epitope reveals critical determinants of breadth of recognition, and structural comparison with other antibodies and ligands defines an inhibitor-binding cavity as a site of immune vulnerability. We utilize the structure to elucidate molecular factors that underlie somatic selection of a critical deletion in the CA45 heavy chain—one that enables antibody recognition of a pre-primed GP ectodomain, and that likely contributes to antibody inhibition of cathepsin loop cleavage. Our analysis reveals structural determinants for near pan-ebolavirus recognition and efficacy by an immunization-induced antibody and lays the groundwork for further development of cross-protective countermeasures.

## Results and Discussion

### Crystal structure of CA45-EBOV GP complex

**Fig. 1:**
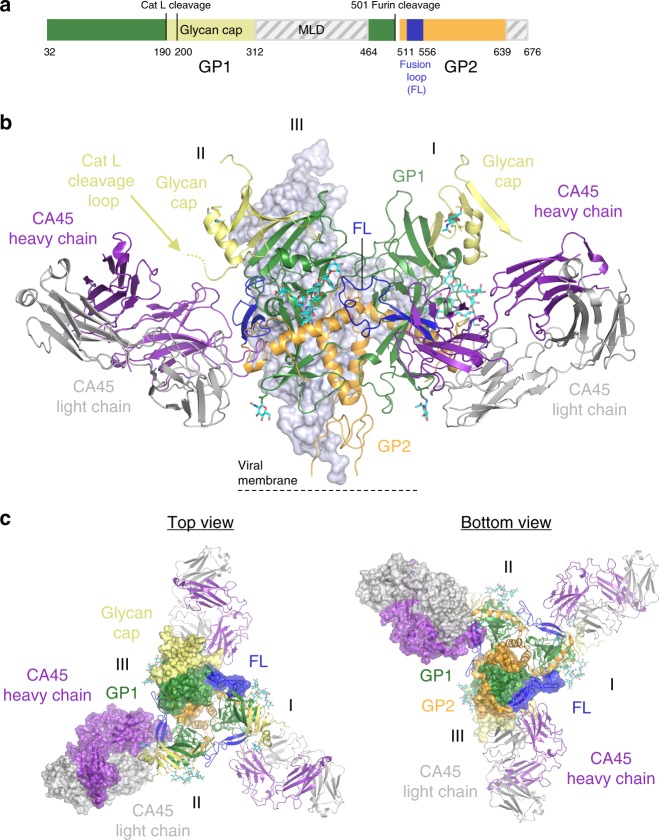
Crystal structure of EBOV GPΔMuc bound to CA45 Fab. **a** Schematic of the EBOV GPΔMuc construct used in crystallizations, with the mucin-like domain (MLD) and transmembrane (TM) domain deleted (gray hatch). **b** Side view of crystal structure of trimeric EBOV GPΔMuc (GP1, green, with glycan cap yellow; GP2, orange, with fusion loop (FL) blue) bound to CA45 Fab (heavy chain purple; light chain gray). Protomer III in the back is shown in light blue surface representation for clarity. **c** Top and bottom views of structure in **b**, shown in combined surface and cartoon representations with all protomers colored by subunit and domain

The GP ectodomain of EBOV strain Mayinga-76 with a deleted mucin-like domain (GPΔMuc) (Fig. 1a and Supplementary Fig. 1a) was expressed transiently in mammalian HEK293S GNTI^−/−^ cells and purified by size exclusion chromatography (SEC). The purified glycosylated GPΔMuc protein was incubated with the antigen-binding fragment (Fab) of antibody CA45, and the resulting complex separated and further purified by SEC. The CA45 Fab-EBOV GPΔMuc complex was set up in a panel of sitting drop vapor diffusion crystallization screens, yielding crystals that in one condition diffracted X-rays to 3.72 Å (Fig. 1a,b and Table 1). Indexing and processing revealed a rhombohedral crystal lattice, in space group H32. The structure was solved with molecular replacement using the unliganded structure of EBOV GP (PDB ID 5JQ3) and the structure of a macaque antibody (PDB ID 3Q6G) as search models^20^. One CA45 Fab-GP protomer complex was present in each asymmetric unit, yielding three CA45 Fabs per GP trimer (Fig. 1b, c). Ordered electron density corresponding to both GP subunits and to the CA45 Fab was observed and utilized for model building, although density for the constant region of the antibody was less well ordered, most notably for the heavy chain (Supplementary Fig. 1b).

**Table 1:** Data collection and refinement statistics

	EBOV GP∆Muc+CA45 Fab
*PDB ID*	6EAY
*Data collection*	
Space group	H32
Cell dimensions	
*a*, *b*, *c* (Å)	153.4, 153.4, 335.9
*α*, *β*, *γ* (°)	90, 90, 120
Resolution (Å)	76.71–3.72 (4.07–3.72)^a^
*R*_merge_	0.181 (0.589)
*I* / *σI*	9.9 (3.9)
*R*_pim_	0.054 (0.176)
CC_1/2_	0.993 (0.859)
Completeness (%)	87.2 (88.5)
Redundancy	9.8 (9.7)
*Refinement*	
Resolution (Å)	45.1–3.72 (4.01–3.72)
No. reflections	14,037 (1402)
*R*_work_/*R*_free_	25.79/29.71
No. atoms	5376
Protein	5254
Ligand/ion	122
Water	–
*B*-factors	112.5
Protein	112.5
Ligand/ion	111.9
Water	–
R.m.s. deviations	
Bond lengths (Å)	0.002
Bond angles (°)	0.76

^a^Values in parentheses are for highest-resolution shell

The CA45 antibody in the present structure approached GP from the side, similar to other base-binding antibodies that target the equatorial surface of GP and consistent with the reported 11 Å EM reconstruction (Fig. 1b, c)^7,8,16,17^. Structural comparison of CA45-bound EBOV GPΔMuc with unliganded and NPC1-C receptor-bound GPs revealed CA45 recognized a conformation of GP more similar to the unliganded form (Supplementary Fig. 1c)^11,21^. Several differences with unliganded GP were however observed. These included a shift in strand β1–β2 and a distinct β-strand organization in the glycan cap, which was likely imposed by crystal lattice contacts that displaced strand β18 with strand β17 of a neighboring lattice molecule (Supplementary Fig. 1d). Electron density corresponding to glycan moeities was observed in three out of nine *N*-linked glycosylation sequon sites, two of which were present solely as *N*-acetyl glucosamines. At the third position, N563, electron density corresponding to a full Man-5 glycan was observed (Supplementary Fig. 1e).

### CA45-EBOV GP interactions

**Fig. 2:**
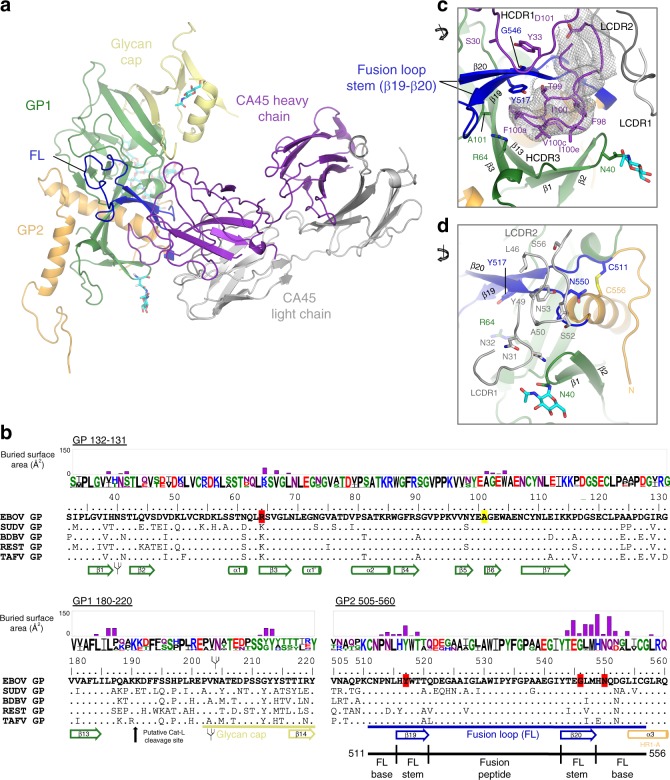
CA45-EBOV GP interactions. **a** Single protomer of EBOV GPΔMuc bound to CA45 Fab, colored as in Fig. 1. **b** Sequence alignment of representative sequences of the five ebolavirus species at selected regions of GP. Buried surface areas on GP residues at the CA45-binding interface are plotted for each residue of the epitope. Residues of EBOV GP that ablate CA45 recognition upon alanine mutation or those associated with viral escape are highlighted in red and yellow, respectively. Sequence logo reflects sequence variation across representative ebolavirus sequences from previous outbreaks^35^, with residues colored by amino acid type. GP subdomains, secondary structure elements, and N-linked glycosylation sequon sites are annotated below the sequence alignment. **c** Close-up view of CA45 interactions with fusion loop stem. Residues sensitive to alanine mutation and viral escape are shown in stick representation on GP along with select interacting CA45 residues. 2fofc composite omit electron density contoured at 1*σ* is shown for the HDCR3 in gray. **d** Close-up view of light chain interactions with fusion loop base

The CA45 epitope on GP observed in the crystal structure was predominantly located in and around the fusion loop stem, made up of strand β19–β20 positioned along the equatorial GP1–GP2 interface of GP. A distinct locus of interaction was also observed between CA45 and N-terminal residues of the GP1 glycan cap (Fig. 2a, b and below). Both the heavy and light chains of CA45, including their complementarity determining regions (CDRs) and framework regions (FRs), mediated interactions across a mostly continuous interface on GP (Fig. 2 and Supplementary Table 1). A total interactive surface of 1105.7 Å^2^ was observed on the antibody with a corresponding interactive surface of 1104.9 Å^2^ observed on GP (Supplementary Table 1). The size of the CA45 interactive surface on GP exceeded those of all other antibodies that target the GP1–GP2 interface of GP for which structures were available for analysis (Supplementary Figs. 2 and 3). In total, 34 residues on GP were present at the interface with CA45, 16 on GP1, and 18 on GP2, corresponding to 32% and 68% of the interface, respectively. Interactions of CA45 with GP thus bridged across both GP1 and GP2 subunits, consistent with a potential mechanism of inhibition described previously for antibody 16F6 (Supplementary Figs. 2 and 3)^16^.

The most extensive interactions between the antibody and GP were mediated by the 23 amino acid CA45 HCDR3 loop, which accounted for ~55% of the total contact interface with GP (Fig. 2a–c and Supplementary Table 1). Apical residues of the HCDR3 loop inserted into the previously defined “DFF” inhibitor-binding pocket^21^, located beneath the β19–β20 fusion loop stem, displacing native GP1 residues D192, F193, and F194, which were disordered in the structure (Fig. 2a, c and below). The CA45 interface with GP extended from the DFF pocket to the outer and upper faces of the β19–β20 fusion loop stem through additional HCDR3 contacts, as well as through contacts mediated by residues of the HCDR1 (Fig. 2c). Light chain FR2 and LCDR2 domains in turn mediated interactions toward the disulfide-bonded base of the fusion loop (Fig. 2d). Although CA45’s interactions with GP1 were predominantly located within the DFF cavity, additional interactions with this subunit were also observed above the fusion loop stem and with the N-terminus of the glycan cap (Fig. 2a, b, d and Supplementary Fig. 2 and below).

Four residues on GP were previously shown to specifically ablate CA45 binding in alanine scan saturation mutagenesis analyses, one on GP1—R64—and three on GP2—Y517, G546, and N550^7^. All four of these residues were observed to lie within the CA45-GP interface in the present structure, with Y517 present within the DFF inhibitor-binding pocket, R64 on the lower outer edge of the pocket, and G546 present on the outer face of the β20 fusion loop strand (Fig. 2b–d and below). Residue N550 was only partially buried by the antibody and its side chain pointed away from the antibody interface, although interactions with its backbone were observed (Fig. 2d). We note that the more limited interactions of CA45 with residue N550 were consistent with previous observations that N550D or N550Q substitutions in EBOV GP had benign effects on CA45 binding^7^.

Previous studies also defined CA45 viral escape variants, which in all cases contained both the A101V and K588R mutations in GP, and in two additional cases also contained the N643D or A654T mutations in the GP2 stalk (not present in the crystallized construct)^7^. Residue A101 in the present structure was found deep within the DFF inhibitor-binding pocket and formed hydrophobic contacts with apical HCDR3 residue F100a (Supplementary Fig. 4). Modeling of the A101V escape mutation at this position predicts a clash would form with CA45 F100a, consistent with disruption of antibody binding and viral escape (Supplementary Fig. 4). Residue K588 was present outside of the CA45-GP interface and its effect on CA45 escape could not be directly explained by the structure. We note that despite the relatively large CA45 epitope on GP defined by the structure, few residues within the epitope itself have thus far been reported to lead to viral escape and appear to arise only with accompanying mutations—possibly indicating a higher bar for viral escape from this antibody.

### Structural basis for broad ebolavirus neutralization by CA45

**Fig. 3:**
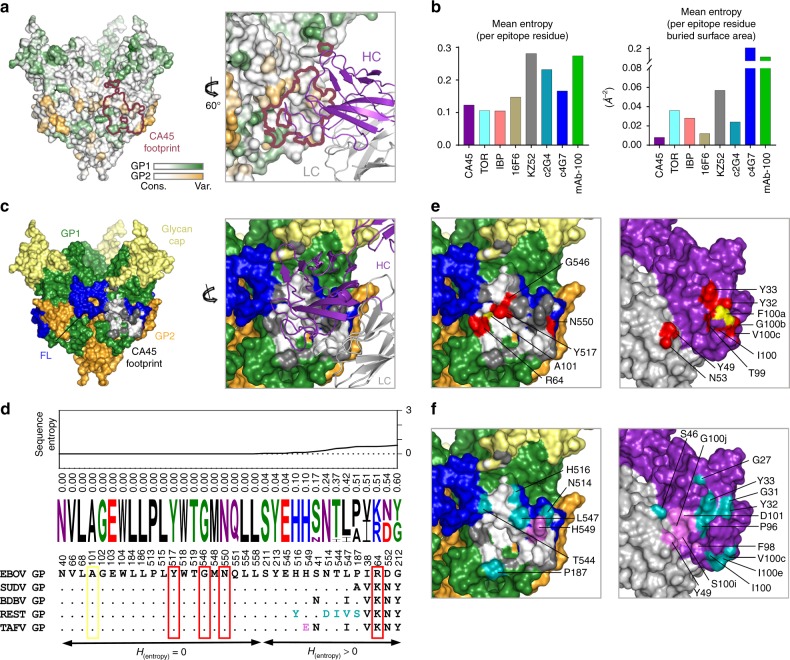
Structural basis for broad ebolavirus neutralization by antibody CA45. **a** Surface representation of CA45-bound EBOV GPΔMuc trimer (left) colored by Shannon sequence entropy on a scale of increasing entropy from white to green (GP1) and white to orange (GP2), with border of CA45 footprint colored raspberry. Close-up view rotated by 60° (right), with bound CA45 heavy and light chains shown in purple and gray, respectively. **b** Comparison of mean entropies of CA45 epitope with those of other neutralizing antibodies and inhibitors that target the GP1–GP2 interface, by mean entropy per GP-binding site residue (left) or normalized by individual residue buried surface area (right). **c** Surface representation of CA45-bound EBOV GPΔMuc trimer (left), colored by subunit and subdomain, with CA45 epitope residues conserved in > 99.5% of ebolavirus isolates colored white and variable residues colored dark gray. Close-up and rotated view of CA45 epitope with bound antibody shown as colored in **a**. **d** Sequence alignment of CA45 epitope residues across representative ebolavirus sequences. Residues are listed in order of increasing sequence entropy, with entropy values listed and graphed above. Sequence logo reflects sequence variation across representative ebolavirus sequences from previous outbreaks^38^, with residues colored by amino acid type. CA45-specific alanine sensitive mutations and escape mutation are boxed in red and yellow, respectively. Residues unique in RESTV and TAFV colored teal and violet, respectively. **e** “Open-book” view of right panel in **c** with surface representation of antibody shown rotated by 180° relative to view in **c**. Alanine sensitive mutations and escape mutation mapped onto surface of GP in red and yellow respectively (left), with corresponding interface residues on antibody colored similarly (right). **f** “Open-book” view of right panel in **c**, with residue positions unique to RESTV and TAFV mapped onto surface of GP in teal and violet, respectively, with corresponding interface residues on antibody colored similarly (right)

The present structure of CA45 bound to GP represents the first reported crystal structure of a GP1–GP2 interface-binding antibody with near pan-ebolavirus neutralizing capacity bound to GP. As such, we sought to establish a structural basis for its cross-recognition and breadth relative to other structurally defined GP1–GP2 interface-binding antibodies and inhibitors^6,8,16–18,21–24^. We compared the Shannon sequence entropies (*H*) of GP residues at the interface with CA45 against those at the interfaces of other antibodies and inhibitors (Fig. 3a, b)^25^. Shannon sequence entropy, a measure of sequence variation, was determined for each GP residue position based on alignment of 236 unique ebolavirus GP sequences determined from a database of 1422 sequences^26^. At the time of writing, this database was composed of 1370 sequences of EBOV GP, 20 of SUDV GP, 7 of BDBV GP, 22 of RESTV GP, and 3 of TAFV (Supplementary Table 2). The lower the sequence entropy of a residue, the more conserved the residue, with an entropy of 0 representing full conservation.

Examination of the sequence entropies of the 34 GP residues of the CA45 epitope revealed an average entropy of 0.12 for each GP residue at the antibody binding interface and an average entropy of 0.008 when normalized by the degree to which each epitope residue’s surface was buried by the antibody (Fig. 3b). These entropy values were lower than those of any other neutralizing antibody analyzed, either as mean entropy per residue or normalized by residue buried surface, consistent with pan-efficacy of the antibody (Fig. 3b). Mean sequence entropies of the binding interfaces of the pan-ebolavirus small-molecule inhibitors toremifene and ibuprofen were ~0.11 per residue each, similar if slightly lower than CA45’s average entropy of 0.12 per residue. When normalized by individual residue buried surface, however, their mean entropies increased relative to CA45’s, due in part to their comparatively smaller binding interfaces (Fig. 3b and Supplementary Figs. 2 and 3 and below).

To further establish the structural basis for breadth of CA45 recognition, we examined sequence variation at each of the 34 CA45 epitope residue positions across the ebolavirus genus (Fig. 3c, d and Supplementary Table 2). Twenty out of 34 epitope residues had sequence entropies of 0 and were fully conserved across all examined ebolavirus GP sequences (Fig. 3c, d). An additional three residue positions (211, 213, 545), which each had entropies of 0.04, differed in only 1 variant each suggesting sequence conservation of > 99.5% at these three positions across the genus (Fig. 3c, d and Supplementary Table 2). The remaining 11 positions of the epitope exhibited more consistent discrete differences between species, with sequence entropies that ranged from 0.10 to 0.60 (Fig. 3c, d and Supplementary Table 2).

Antibody breadth of recognition can be achieved either through phylogenetic conservation of epitope residues, in many cases due to functional or structural constraints, or through antibody tolerance of sequence variation, or through a combination of both. In the case of CA45, we utilized previously reported alanine scan saturation mutagenesis data in the context of EBOV GP^7^ to assess CA45 sensitivity to mutation within both conserved and non-conserved epitope residue positions. Of the four mutations that result in near complete specific ablation of CA45 recognition, R64, Y517, G546, and N550, the latter three are fully conserved across all ebolaviruses, while the former, R64, is strictly mutated only to a phsyicochemically similar lysine across the genus (Fig. 3e). Alanine mutation at three additional epitope residues, E103, L515, and W518, also knocked out CA45 binding, although these positions were also sensitive to control mAb recognition, suggesting they could be important for overall GP integrity or folding^7^. Nevertheless, all three of these residues also belonged to the set of fully conserved epitope residues with zero sequence entropy. Thus, with the exception of R64 (which only varied to a lysine), CA45 epitope residues that were sensitive to alanine mutation were found fully conserved across the genus, potentially due to functional or structural constraints, while mutations at less conserved epitope positions were largely tolerated by the antibody, both factors likely contributing to the antibody’s breadth.

Of the ebolaviruses, CA45 neutralizes BDBV with greatest potency (IC_50_ 0.9–2.8 nM), followed by EBOV (IC_50_ 3–4.4 nM) and SUDV (IC_50_ 3.3–12 nM)^7^. It neutralizes RESTV approximately tenfold less well (IC_50_ 42 nM) and is completely refractory to TAFV neutralization^7^. Although all ebolaviruses were identical in sequence at the 20 epitope residue positions described above, they differed to varying degrees at other epitope positions (Fig. 3c, d and Supplementary Table 2). To assess whether these sequence differences could account for the more pronounced differences in antibody efficacy observed against RESTV and TAFV species, we examined their analogous CA45 interfaces in greater detail. In the case of RESTV, in isolates used for functional analysis, five epitope residue positions—187, 514, 516, 544, and 547—were found to vary to amino acids that were not observed in any other bound species (Fig. 3d, f and Supplementary Table 2). Although none of these five residues led to ablation of CA45 recognition when mutated to alanine in the context of EBOV GP^7^, indicative of at least some degree of antibody tolerance for mutation at these positions, several antibody residues that formed an interface with these positions were in fact sensitive to alanine mutation. These included heavy chain residues G31, Y32, Y33 within the HCDR1 and residue I100e within the HCDR3. The sensitivity of these antibody residues to mutation was therefore consistent with possible relevance of this interface for reduced antibody recognition and efficacy against RESTV—whose GP differs from other ebolaviruses at this interface (Fig. 3f).

In the case of TAFV, which is refractory to CA45 binding and neutralization, seven residues within its analagous CA45 epitope differed from those in EBOV GP (Fig. 3c, d, f). Six of these residues were identical to those found in BDBV—a species bound and neutralized well by CA45^7^. Only a glutamate at position 549 was found to be distinct in TAFV, present as a histidine in all other ebolaviruses. Residue H549 in the current structure is situated to mediate a putative salt bridge with CA45 heavy chain residue D101—an antibody residue that is highly sensitive to alanine mutation in the context of EBOV, SUDV, and BDBV GP (Fig. 3f and Supplementary Fig. 5)^7^. Although an acidic H549E substitution, as present in TAFV GP, would be expected to disrupt this salt bridge, experimental evidence indicates that neither H549E nor H549A substitutions in the context of EBOV GP abrogate CA45 binding (Supplementary Fig. 5)^7^. Thus, although the absence of this salt bridge in the context of TAFV may contribute to loss of antibody binding, there are likely additional factors at play within TAFV that preclude CA45 recognition.

### Comparison with other neutralizing antibodies and inhibitors

**Fig. 4:**
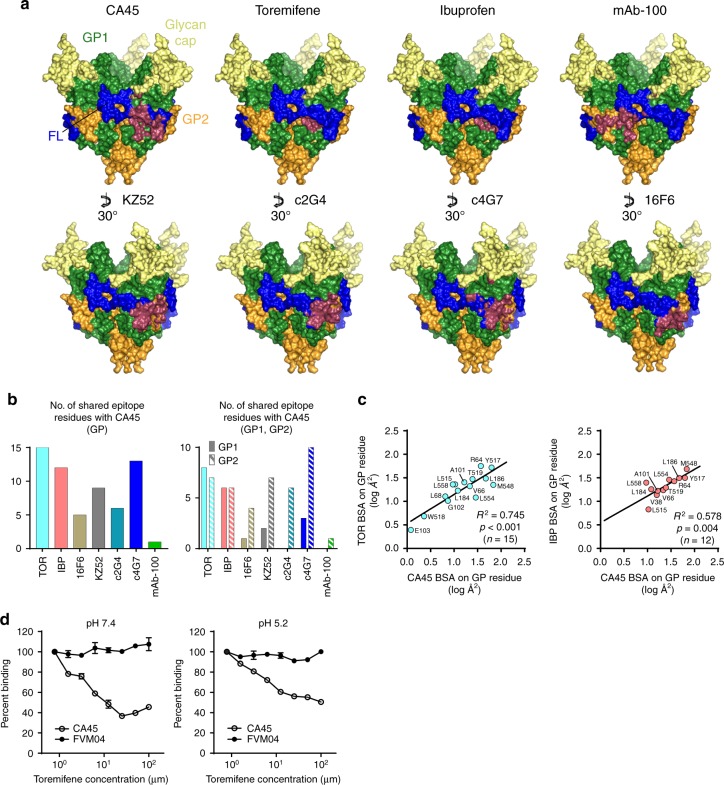
Comparison with other neutralizing antibodies and inhibitors that target the GP1–GP2 interface. **a** Epitope footprints of antibodies and small-molecule inhibitors that target the GP1–GP2 interface mapped onto the surface of the CA45-bound EBOV GPΔMuc trimer. Footprints of each respective ligand colored raspberry. Lower panels rotated by 30° relative to CA45 panel. **b** Comparison of number of shared epitope residues with CA45, total (left) and by GP subunit (right)**. c** Pairwise linear regressions of log transformed buried surface areas (BSA) of shared GP interface residues of CA45 with toremifene (TOR; left) and ibuprofen (IBP; right). Statistical correlations were determined under the null hypothesis of zero correlation against the alternative hypothesis of non-zero correlation. *R*^2^, coefficient of determination; *p*, *p*-value; *n*, sample size. **d** ELISA competition analysis of toremifene against neutralizing antibodies CA45 and FVM04 for binding to recombinant EBOV GPΔMuc, at pHs 7.4 and 5.2. Data were normalized to percent binding relative to no competitor control and error bars represent SD of sample duplicates from plotted mean

To further pinpoint residues and domains on ebolavirus GP that distinguished CA45 recognition, we investigated the degree to which its binding site on GP overlapped or diverged from the binding sites of other antibodies and inhibitors that target the GP1–GP2 interface. We utilized available structures of GP in complex with antibodies c4G7, c2G4, KZ52, 16F6, and mAb-100, and in complex with the pan-reactive small-molecule inhibitors toremifene and ibuprofen (Fig. 4a, b and Supplementary Fig. 2)^6,8,16–18,21–24^. This analysis revealed that the CA45-binding site on GP shared the most residues in common with the binding site of the small-molecule inhibitor toremifene, overlapping at 15 residues, followed by overlap with the binding site of the ZMapp antibody c4G7, overlapping at 13 GP residues (Fig. 4a, b). Overlap with the binding sites of the other ligands ranged from 12 (ibuprofen) to only 1 residue (mAb-100) (Fig. 4a, b). Although the shared interface residues of CA45 and the small-molecule inhibitors were split roughly equally across GP1 and GP2, the shared interface residues with the other antibodies were predominantly localized to GP2 (Fig. 4b).

To quantitatively assess the relationship between the CA45 epitope on GP with those of the other antibodies and ligands, we undertook a pairwise regression analysis of the buried surface areas of their shared GP interface residues (Fig. 4c, Supplementary Fig. 2, and Supplementary Table 3). Although partially dependent on the differing resolutions of the available structures (ranging from 2.7-6.7 Å) and the number of intersecting interface residues, pairwise linear regressions of the shared residue log transformed buried surface areas yielded statistically significant positive correlations between the GP-binding sites of CA45 and the inhibitors toremifene and ibuprofen, with *p*-values < 0.0001 and 0.0041, respectively (Fig. 4c, Supplementary Fig. 2, and Supplementary Table 3). Statistically significant relationships were not observed with antibody c4G7, nor with any other antibody analyzed (Supplementary Fig. 6 and Supplementary Table 3).

To determine whether the intersection in binding sites of CA45 and the small-molecule inhibitors could be manifested experimentally, we undertook an enzyme-linked immunosorbent assay (ELISA) competition binding assay between CA45 and the more potent inhibitor toremifene. Concentrations of toremifene ranging from 100 to 0.78 μM were premixed with a constant amount of CA45 IgG and then incubated with captured recombinant EBOV GPΔMuc. Experiments were performed at both pH 7.4 and 5.2, as well as with a control antibody FVM04 that recognizes a conformational epitope in the receptor-binding region of GP^5,19^. As shown in Fig. 4d, toremifene was able to effectively compete with CA45 for binding to GP with IC_50_s of 6.2 and 5.5 μM at pHs 7.4 and 5.2, respectively. Toremifene did not inhibit binding of antibody FVM04 to GP, at either pH, suggesting its effects were specific to the CA45-binding site and did not extend more globally to the receptor-binding region of GP (Fig. 4d).

### GP inhibitor-binding cavity as a site of immune vulnerability

**Fig. 5:**
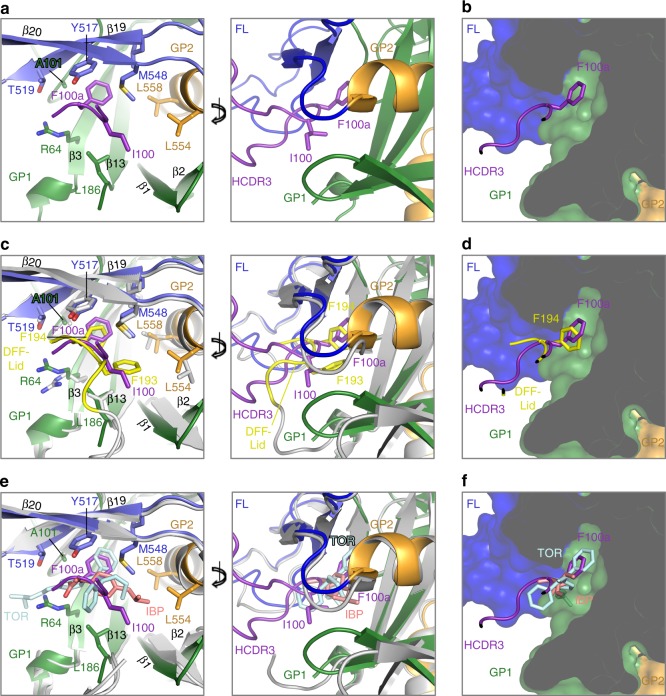
CA45 defines an inhibitor-binding cavity on GP as a site of immune vulnerability. **a** The CA45 HCDR3 loop (purple) inserts into the DFF inhibitor-binding cavity located beneath strand β19–β20 of the fusion loop stem (blue). GP1 is colored green, GP2 orange, and fusion loop (FL) blue. Cartoon representation of front of cavity with apical residues of HCDR3 shown (left) and rotated with a more extensive view of HCDR3 (right). **b** Cross-sectional view of right panel in **a**, with GP shown in surface representation to reveal DFF cavity. **c** Superposition of unliganded GP (gray; PDB ID 5JQ3) onto CA45-bound GP (representations in left and right panels are as described in **a**). Residues of the “DFF lid” of unliganded GP1 are shown in yellow, with residues F193 and F194 shown in stick representation. **d** Cross-sectional view of right panel in **c**, with GP shown in surface representation. **e** Superposition of toremifene- and ibuprofen-bound GP structures (gray; PDB IDs 5JQ7 and 5JQB, respectively) onto CA45-bound GP structure, with toremifene (TOR) shown in pale cyan and ibuprofen (IBP) shown in salmon (representations in left and right panels are as described in **a**). **f** Cross-sectional view of right panel in **e**, with GP shown in surface representation

Given the intersection in the GP-binding sites of CA45 and the small-molecule inhibitors, as well as their competitive binding, we compared their respective structural modes of interaction with GP (Fig. 5). Toremifene and ibuprofen, as well as others that belong to a larger class of estrogen receptor modulators, bind within the DFF cavity and are reported to inhibit virus–cell membrane fusion by destabilizing GP in a pH-dependent manner, possibly by premature triggering^21,23,24,27,28^. In unliganded GP, the DFF cavity is occupied by its namesake GP1 residues D192, F193, and F194 that create a lid (the “DFF lid”) that protects hydrophobic residues that line the cavity^21^. The DFF lid is expelled from the cavity upon binding of the inhibitors.

In the present structure, we observed that binding of CA45 to GP and insertion of its HCDR3 into the DFF cavity also displaced the native GP1 D192, F193, and F194 residues, which were disordered in the structure (Fig. 5a, b). Superposition of the unliganded structure of GP onto the CA45-bound structure revealed remarkable molecular mimicry on the part of the antibody in its interactions within the cavity. As shown in Fig. 5c, d the mode of binding of CA45 HCDR3 apical residues I100 and F100a was highly similar to that of native DFF lid GP1 residues F193 and F194 within the cavity, especially when comparing placement of the aromatic side chains of F194 of GP1 and F100a of CA45 (Fig. 5c, d). Consistent with this observation, alanine substitution of F100a in CA45 has previously been shown to lead to nearly complete loss of antibody binding to EBOV, SUDV, and BDBV GP^7^. Superposition of the structures of inhibitor-bound GP onto the CA45-bound structure also revealed remarkable overlap in placement of apical residues of the CA45 HCDR3 and the small-molecule inhibitors, more markedly for toremifene (Fig. 5e, f). Indeed, CA45 contacted many of the same set of residues contacted by the inhibitors within this cavity (Fig. 4c and Supplementary Fig. 2).

These results implicate the DFF pan-ebolavirus inhibitor-binding cavity as a bona fide site of broad immune vulnerability on GP. Saturation mutagenesis analyses previously identified residues R64, Y517, and G546, as necessary for CA45 binding, and we observe all them at the CA45 interface near or within the DFF cavity (Figs. 3e and 5). Likewise, residue A101, which was shown to be associated with viral escape from CA45, was found deep within the DFF cavity and formed interactions with the apex of the CA45 HCDR3 (Figs. 3e and 5 and Supplementary Fig. 4)^7^. We note that residues L554 and L558 that line the DFF cavity in the prefusion state of GP, and form interactions with the DFF lid, with the inhibitors, and with CA45, are found at the N-terminus of the N-heptad helix of the six helix bundle in postfusion GP2, partially buried within its core (Supplementary Fig. 7)^29^. These and other interactions of CA45 within the DFF cavity and additional interactions along the outer and upper faces of the fusion loop stem may interfere with proper fusion loop release or transition of GP to the postfusion state. We note that although the overall architecture of the cavity is present within marburgviruses, significant differences are observed both in residues within the cavity as well as in structural domains that protect it, which both likely contribute to lack of CA45 recognition of marburgvirus GP (Supplementary Fig. 8).

### Structural determinants for somatic selection of CA45

**Fig. 6:**
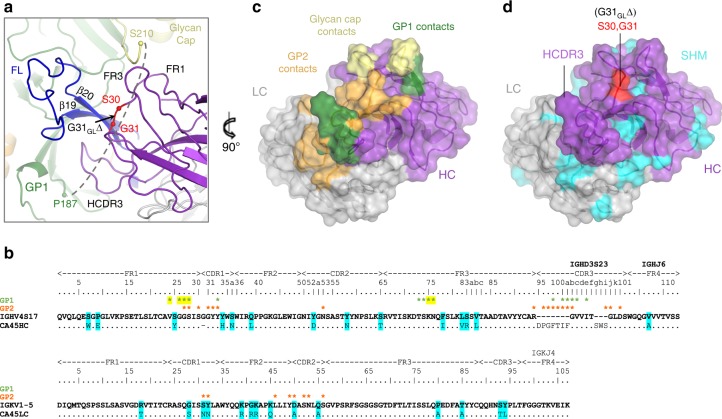
Somatically matured CA45 bridges the termini of the cathepsin cleavage loop. **a** A close-up view of CA45 bound to EBOV GPΔMuc, with Cα atoms of residues that flank germline deletion G31_GL_Δ shown in red spheres. CA45 mediates interactions with both termini (green and yellow spheres) of the disordered cathepsin loop (dashed line). **b** Sequence alignment of mature CA45 heavy and light chains against cynomolgus macaque germline precursors. Residues that undergo somatic mutation in CA45 are shaded in cyan and those that are buried at the interface with GP1 or GP2 are labeled with green and orange asterisks, respectively. Asterisks shaded yellow mediate interface with glycan cap. **c** CA45 paratope with full GP interface mapped onto CA45 Fab shown in surface representation (heavy chain, purple; light chain, gray), rotated by 90° from view in **a**. Footprints of GP1 are shown in green, of glycan cap yellow, and of GP2 orange. **d** CA45 residues that are somatically hypermutated (SHM) are mapped onto CA45 surface in cyan with unmutated heavy and light chain residues colored in purple and gray, respectively. Shown in same orientation as in **c**. Residues that flank the G31_GL_Δ germline deletion are shown in red

CA45 is encoded by cynomolgus macaque heavy chain germline genes IGHV4S17, IGHD3S23, and IGHJ6, and light chain germline genes IGKV1-5 and IGKJ4^7^. Its VH and VL genes are somatically mutated by 9.9% and 7.5% on the nucleotide level, respectively (~14% each on the amino acid level), and the VH region has a deletion of germline residue G31_GL_ within the HCDR1 (Fig. 6). Inferred germline reversion analyses of these precursor genes has previously shown that heavy and light reversion (CA45-H_GL_-L_GL_) leads to loss of binding of CA45 to GPΔmuc but not to cleaved GPcl^7^. This phenotype was shown to be caused by the G31_GL_ deletion in the HCDR1 in that its re-insertion into mature CA45 ablates binding to GPΔmuc but not to GPcl^7^.

To address the structural basis for somatic selection of this deletion in the CA45 heavy chain, we examined its placement in the structure (Fig. 6). Residue G31_GL_ lies immediately downstream of heavy chain FR1, which, in conjunction with heavy chain FR3, forms a cradle that interacts with the N-terminus of the glycan cap. In the absence of a deletion at G31_GL_, the FR1 loop that forms part of this cradle would likely extend into the glycan cap and disrupt antibody binding—a disruption that would not occur in the context of GPcl that lacks a glycan cap. Thus, selection of a deletion at G31_GL_ was likely necessitated to accommodate antibody binding to an uncleaved ectodomain with an intact glycan cap.

By enabling the interaction between CA45 and the full GP ectodomain, the deletion of germline residue G31_GL_ may have also facilitated additional CA45 functions. Namely, CA45 has been suggested to partially inhibit host endosomal cathepsin proteolytic cleavage of GP1, which is required for NPC1-C receptor binding and entry^7^. The cathepsin cleavage loop of GP spans roughly 20 residues of GP1 (188–210) and connects the undercarriage of GP1, beneath the fusion loop stem, to the glycan cap domain (Fig. 6a). This loop is cleaved by host cathepsins upon virus trafficking to late endosomes, and although residues 188–209 of the cleavage loop were disordered in the present structure, both of its termini were found closely associated with the antibody (Fig. 6a)^30,31^. GP1 residues L184, L186, and P187 that lead into the loop formed an interface with the HCDR3 of the antibody, while at the opposite end, antibody heavy chain FR1 and FR3 regions interacted with residues 211–213 of the glycan cap (Fig. 6a–c). The latter interactions, as noted above, were likely enabled by deletion of germline residue G31_GL_. CA45 thus directly bridged both ends of the cathepsin loop, positioning the antibody to contribute to its reported hindrance of loop cleavage^7^.

Taken together, our results define a highly conserved epitope on the fusion loop of ebolavirus GP, at the GP1–GP2 interface, through which broad antibody-mediated neutralization of ebolaviruses can occur. Phylogenetic conservation of critical residues of the epitope, as well as apparent tolerance for mutation elsewhere, are consistent with antibody breadth and lower likelihood for viral escape. The structural definition of residues on ebolavirus GP that confer cross-protection, as elucidated here, provides a template for design of vaccines and immunotherapeutics aimed at conferring similar protective breadth. The concurrent dependence on moieties in both GP1 and GP2 for recognition suggests this target may have the additional benefit of circumventing diversion from soluble forms of GP secreted during active infection.

## Methods

### Protein expression and purification

A codon optimized gene corresponding to Zaire EBOV strain Mayinga-76 GP (EBOV GPΔMuc) was synthesized through an outside vendor (Genscript, Inc.) (Supplementary Fig. 1a). The construct possessed deletions of its mucin-like and transmembrane domains, an added leader sequence, along with C-terminal strep and polyhistidine tags. The gene was subcloned into vector pcDNA3.1 for transient expression in mammalian cells.

Transient expression of EBOV GPΔMuc was undertaken in HEK293S GNTI^−/−^ (ATCC) cells using 293fectin transfection reagent according to manufacturer’s guidelines (Thermo). EBOV GPΔMuc was in all cases co-transfected with a furin expression construct at 30% ratio. Secreted GP protein was harvested from supernatants and purified using Complete His Purification Resin (Roche) followed by Streptactin Resin purification (IBA Biosciences). The GP protein was further purified by SEC Superdex 200 HiLoad 16/600 column in 150 mM NaCl, 2.5 mM Tris-Cl pH 7.5 and 0.02% NaN_3_.

CA45 IgG was expressed in HEK-293F (ATCC) cells by co-transfecting its heavy and light chain expression plasmids using 293Fectin and Freestyle media (Thermofisher). The IgG was purified from supernatants using Protein A Resin (Roche) and eluted using a low pH Protein A elution buffer (Pierce) followed by immediate neutralization with Tris base pH 9.0. CA45 Fab was produced from purified IgG using papain cleavage (Pierce).

### EBOV GPΔMuc-CA45 Fab complex crystallization

CA45 Fab was added to GP at 1.1-fold molar excess and purified by SEC. Protein complex composition was confirmed by SDS polyacrylamide gel electrophoresis. Crystallization of the complexes was undertaken by initial robotic screening using 576 conditions from commercially available screens (Hampton Research; Rigaku Reagents). Crystallization trays were set up using a Mosquito Nanoliter Liquid Handling System (TTP Labtech). Crystals were obtained in a condition made up of 1 M Potassium Sodium Tartrate, 0.1 M MES pH 6.0, and were manually optimized for synchrotron data collection.

### Structure determination and analysis

X-ray diffraction data were processed with the XDS Software Package^32^. Frames from nine crystals were merged with CCP4 Blend^33^. The structure was solved by molecular replacement using Phaser with PDB ID 5JQ3 as a search model for GP and PDB ID 3Q6G as a search model for the antibody^20^. The structure was refined using Phenix^34^ with iterative model building in Coot^35^ utilizing 2fofc, fofc, and composite omit electron density maps. Interactive buried surface areas were determined using Pisa^36^. All graphical images were prepared with Pymol (PyMOL Molecular Graphics System). X-ray diffraction data were collected at SER-CAT ID-22 beamlines of the Advanced Photon Source (Argonne, IL).

Figures of footprints of antibodies and inhibitors onto CA45-bound GP were determined by superimposing their corresponding GP structures (PDB IDs: 5JQ7, 5JQB, 3CSY, 5KEL, 5KEN, 5FHC, 3S88) onto CA45-bound GP. Mapping of atoms on GP within 4.5 Å of the docked antibodies and inhibitors was undertaken with Pymol.

GP buried surface areas by residue for antibodies and inhibitors were determined in Pisa solely using coordinates of their respective PDB IDs (5JQ7, 5JQB, 3CSY, 5KEL, 5KEN, 5FHC, 3S88)^36^. Statistical correlations between buried surface areas (log transformed) of shared epitope residues of CA45 and other antibodies and inhibitors were determined by testing the null hypothesis that they were independent. The null hypothesis was that the correlations were zero (no correlation), and the alternative hypothesis that correlations were thus non-zero. For the four antibodies tested against CA45, the results did not reject the null hypothesis. For the two inhibitors, the null hypothesis was rejected by a significant *p*-value, supporting the possibility they had a positive correlation with CA45. Statistical tests and results for this analysis were generated in Prism and are shown in Supplementary Table 3.

### Shannon sequence entropy analysis and sequence conservation

A manually curated protein sequence alignment containing 236 unique sequences was obtained using sequences from https://www.ncbi.nlm.nih.gov/genomes/VirusVariation/Database/nph-select.cgi?cmd=database&taxid = 186536. The Shannon entropy for each residue position in the alignment was determined using the formula (*H* = −∑ *p*_i_ log_2_*p*_i_) where *p*_i_ is the probability of each amino acid in a column in the alignment^25^. Only amino acids were considered in computing the Shannon entropies. Sequence entropies were mapped onto the GP trimer in Pymol by replacing B-factors with Shannon entropy. Mean entropy per residue BSA was calculated by dividing each epitope residue’s entropy value by its respective antibody buried surface area, followed by mean calculation. Sequence logos were produced using WebLogo3^37^ with a set of 21 unique sequences representing outbreaks (10 EBOV, 7 SUDV, 2 BDBV, 1 RESTV and 1 TAFV) obtained from the Los Alamos HFV/Ebola Database (https://hfv.lanl.gov/content/sequence/NEWALIGN/align.html).

### Competition ELISA assay

EBOV GPΔMuv at 200 ng per well in 100 μL of phosphate-buffered saline (PBS) pH 7.4 was incubated overnight at 4°C in Nunc MaxiSorp Plates. The plates were washed and then blocked in PBS pH 7.4 supplemented with 5% fetal bovine serum and 2% non-fat dry milk powder (Difco) for 1 h at room temperature. For the pH 7.4 ELISA assay, all subsequent washes were undertaken with wash buffer composed of PBS pH 7.4 supplemented with 0.05% tween-20. For the pH 5.2 ELISA assay, all subsequent washes were undertaken with wash buffer composed of 0.05 M sodium acetate pH 5.2, 150 mM sodium chloride, 0.05% tween-20. A series of 1:1 toremifene dilutions were prepared in a separate non-binding 96 well plate in either PBS pH 7.4 with 10% dimethylsulfoxide (DMSO) or in 0.05 M sodium acetate pH 5.2, 150 mM sodium chloride with 10% DMSO. In all cases, a constant amount of antibody at 0.4 μg/mL was present. 100 μl of the mixture was transferred to the GP-captured wells in each corresponding pH. After 1 h, the plates were washed and incubated for 1 h with a 1:2500 dilution of horseradish peroxidase-conjugated goat anti-human secondary antibody (Jackon ImmunoResearch #109-035-003) in 100 µL blocking buffer per well. The ELISAs were resolved using Bio-Rad TMB ELISA substrate and stopped using 1 N sulfuric acid.

## Electronic supplementary material

Supplementary Information

## Data Availability

Structural coordinates and structure factors have been deposited in the RCSB Protein Data Bank under PDB ID 6EAY.
