# Supplementary material for: Structural basis for broad neutralization of ebolaviruses by an antibody targeting the glycoprotein fusion loop
Source: Nat Commun. 2018 Sep 26;9:3934. doi: 10.1038/s41467-018-06113-4 (PMC6158212; doi:10.1038/s41467-018-06113-4)
Supplement: Supplementary file 1 — Supplementary Information [file 41467_2018_6113_MOESM1_ESM.pdf]

Supplementary Information

Structural basis for broad neutralization of ebolaviruses by an antibody targeting the glycoprotein fusion loop

Janus, B.M. *et al.*

a

SIPLGVIHNSTLQVSDVDKLVCRDKLSSTNQLRSVGLNLEGNGVATDVPSATKRWGFRSGVPPKVVNY
EAGEWAENCYNLEIKKPDGSECLPAAPDGIRGFPRCRYVHKVSGTGPCAGDFAFHKEGAFFLYDRLAS
TVIYRGTTFAEGVVAFLILPQAKKDFFSSHPLREPVNATEDPSSGYYSTTIRYQATGFGTNETEYLFE
VDNLTYVQLESRFTPQFLLQLNETIYTSGKRSNTTGKLIWKVNPEIDTTIGEWAFWETKKNLTRKIRS
EELSFTVVNTHHQDTGEESASSGKLGLITNTIAGVAGLITGGRRTRREAIVNAQPKCNPNLHYWTTQD
EGAAIGLAWIPYFGPAAEGIYTEGLMHNQDGLICGLRQLANETTQALQLFLRATTELRTFSILNRKAI
DFLLQRWGGTCHILGPDCCIEPHDWTKNITDKIDQIIHDFVDKTLPDGENLYFQSGSAWSHPQFEKHH
HHHHHH

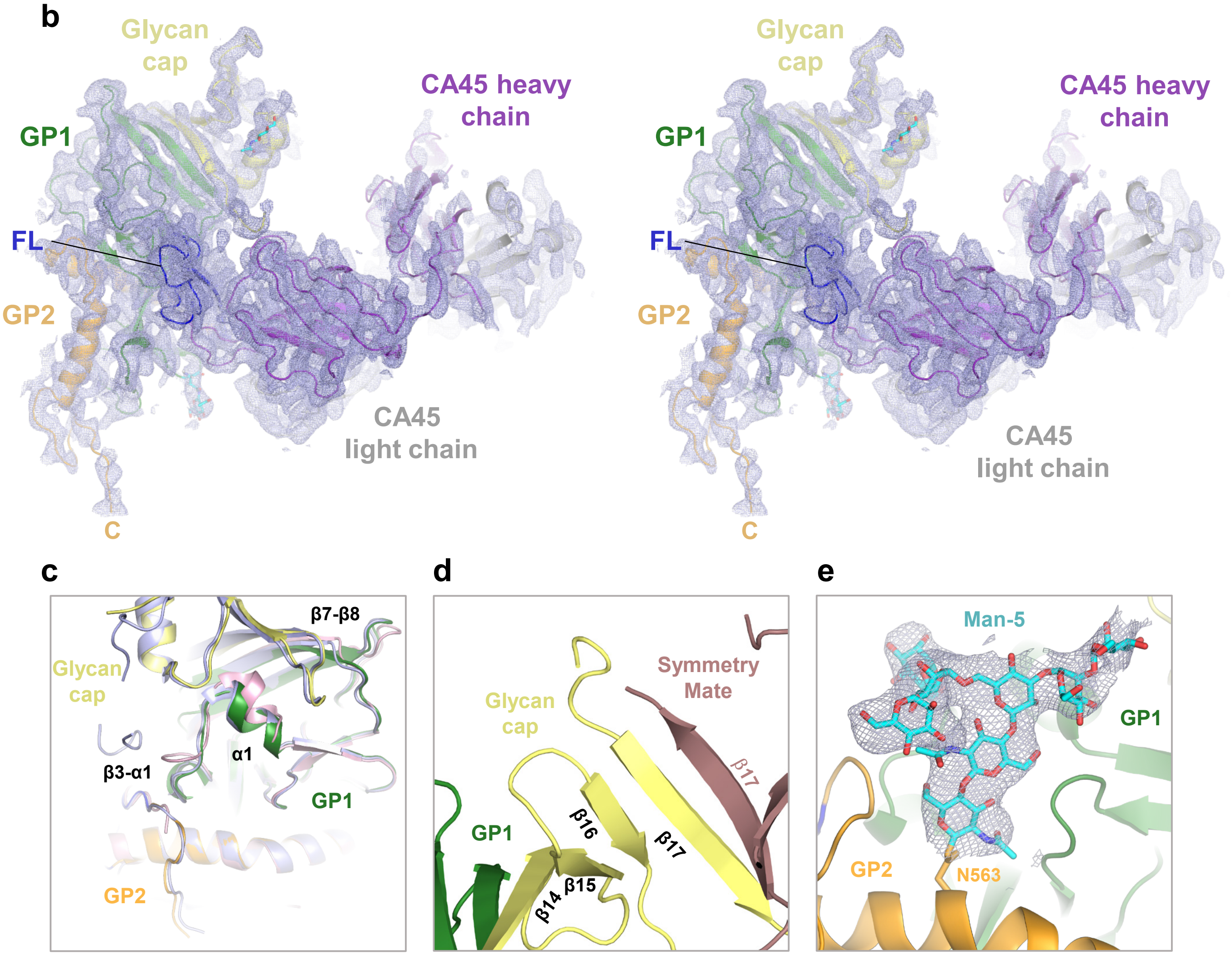

Supplementary Figure 1. Structural features of CA45-bound EBOV GPΔMuc. a. Amino acid sequence of crystallized EBOV GPΔMuc construct. GP1 (green), glycan cap (yellow), GP2 (orange), TEV protease site (blue), strep tag (dark blue), and his-tag (brown). **b.** Stereo image of one GP protomer bound by CA45 Fab shown with 2fofc electron density map (blue-gray) contoured at 1σ. GP1 (green), glycan cap (yellow), GP2 (orange), fusion loop (blue), glycans (cyan), CA45 heavy chain (purple) and light chain (gray). **c.** Structural comparison of CA45-bound EBOV GPΔMuc with NPC1-C receptor-bound and unliganded EBOV GP. A single protomer of CA45-bound EBOV GPΔMuc was superimposed with NPC1-C-bound GPcl (5JQB; pink) and unliganded EBOV GPΔMuc (5JQ3; light blue). The NPC1 bound GPcl has three distinct conformational shifts relative to unliganded GP,as labeled in panel. **d.** Glycan cap β-strand configuration in CA45-bound EBOV GPΔMuc structure (yellow) shown with a neighboring lattice contact (bronze). **e.** A Man-5 glycan chain (cyan) is observed at position N563 of GP2 (orange), shown with 2fofc electron density map (blue-gray) contoured at 1σ.

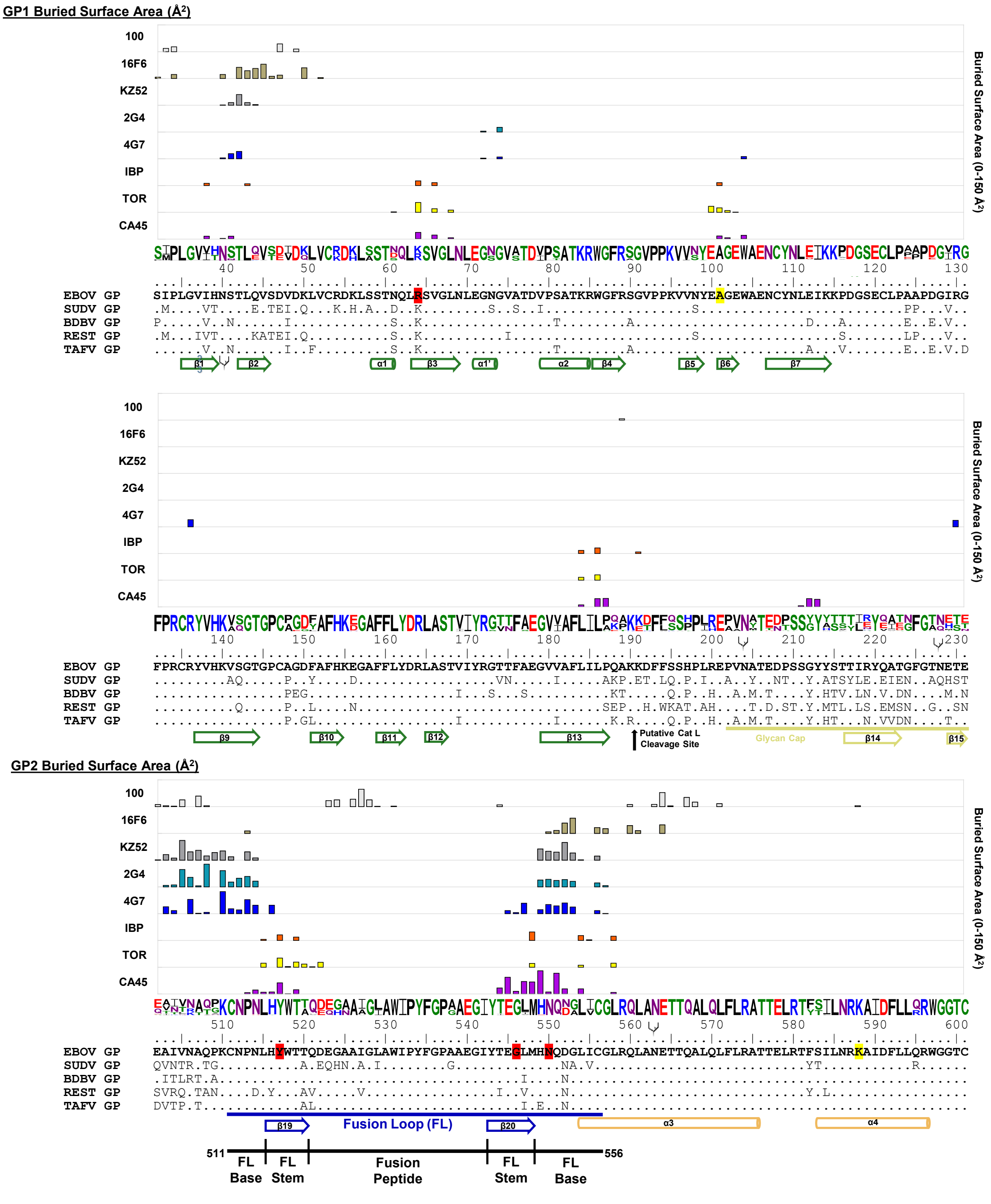

Supplementary Figure 2. Comparison of GP binding interfaces of CA45 versus other neutralizing antibodies and inhibitors. Buried surface areas on GP (determined from PDB IDs 5JQ7, 5JQB, 3CSY, 5KEL, 5KEN, 5FHC, and 3S88) are plotted as bars above each sequence residue (scale for each is 0-150 Å^2). Sequence residues highlighted in red in main sequence alignment ablate CA45 binding when mutated to alanine, and those highlighted in yellow contribute to viral escape from CA45. Sequence logo reflects sequence variation across representative ebolavirus sequences from previous outbreaks, with residues colored by amino acid type. GP subdomains and secondary structure elements are annotated below the sequence alignment. TOR, toremifene; IBP, ibuprofen.

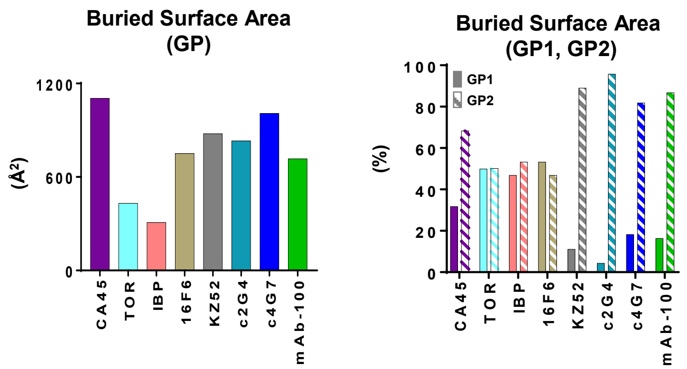

Supplementary Figure 3. Buried surface areas on GP of GP1-GP2 interface-targeting antibodies and inhibitors. Comparison of buried surface areas on GP, total (left) and by GP subunit (right), calculated from respective PDB IDs (as shown and listed in Supplementary Fig. 2). TOR, toremifene; IBP, ibuprofen.

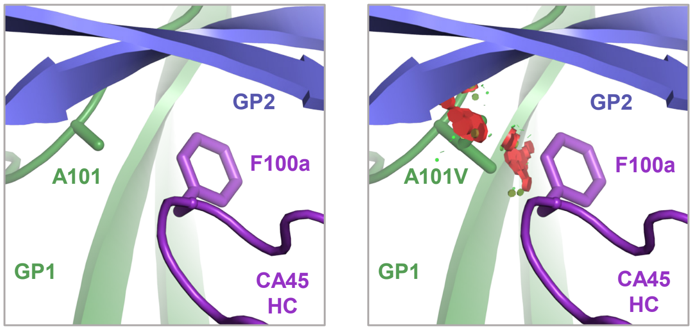

Supplementary Figure 4. Viral escape mutation A101V. Closeup view of interactions between CA45 heavy chain residue F100a and GP1 residue A101 (left). Viral escape variants from CA45 all contain mutation A101V, which as shown modeled on right leads to clashes with F100a (represented as red discs).

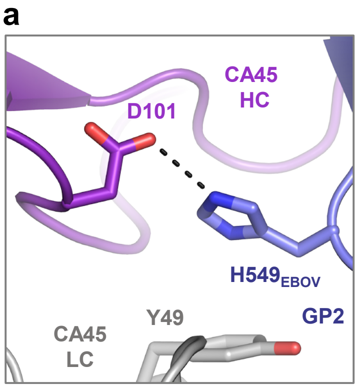

Supplementary Figure 5. Modeling of TAFV residue E549 into CA45-bound EBOV GP. **a.** CA45 heavy chain residue D101 forms a putative salt bridge with residue H549 in EBOV GP. **b.** Residue 549 in TAFV is an E, shown modeled into CA45-bound EBOV GP structure. An H549E substitution would abrogate the salt bridge.

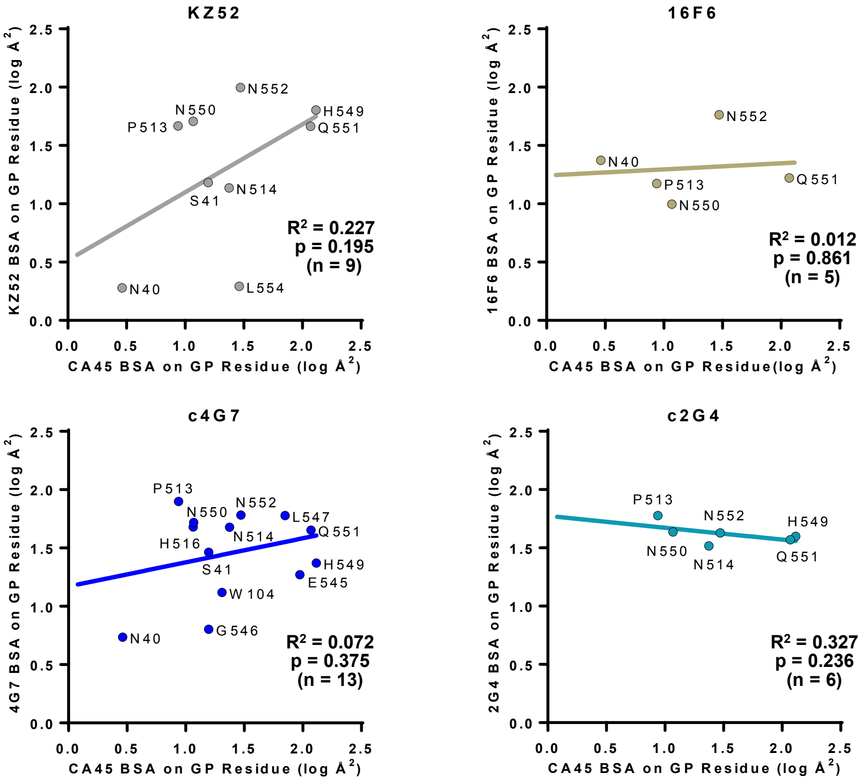

Supplementary Figure 6. Relationship between shared epitope residues of CA45 and other GP1-GP2 interface binding antibodies. Shown are pairwise linear regressions of log buried surface areas (BSA) of shared GP epitope residues between CA45 and antibodies KZ52, 16F6, c4G7 and c2G4 (see Supplementary Table 3). Statistical correlations were determined under the null hypothesis of zero correlation against the alternative hypothesis of nonzero correlation. R^2, coefficient of determination ; *p*, p-value; *n*, sample size.

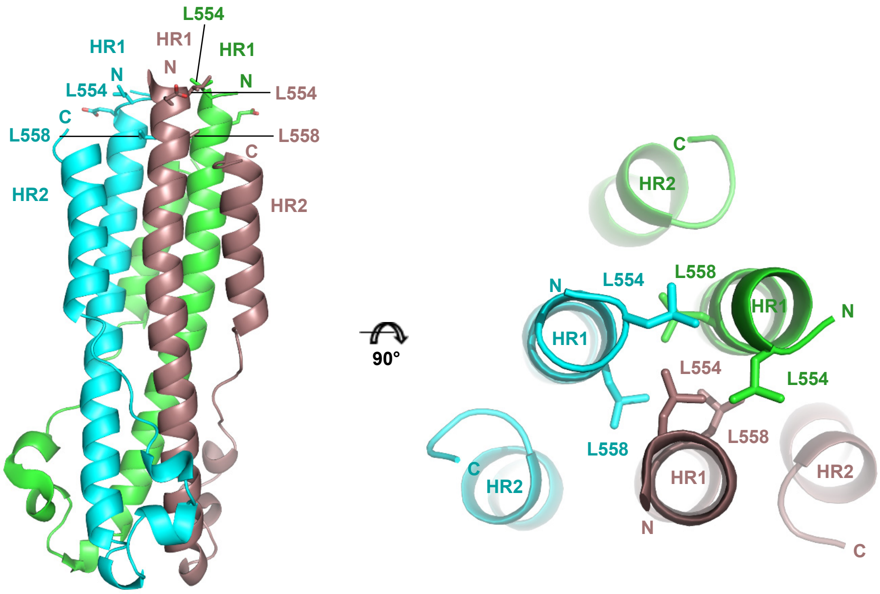

Supplementary Figure 7. Location of DFF cavity residues in postfusion structure of EBOV GP2. GP2 residues L554 and L558 which line the DFF cavity in the prefusion state of GP are partially buried at the N-terminus of the N-heptad repeat core in postfusion GP2 (PDB ID 1EBO).

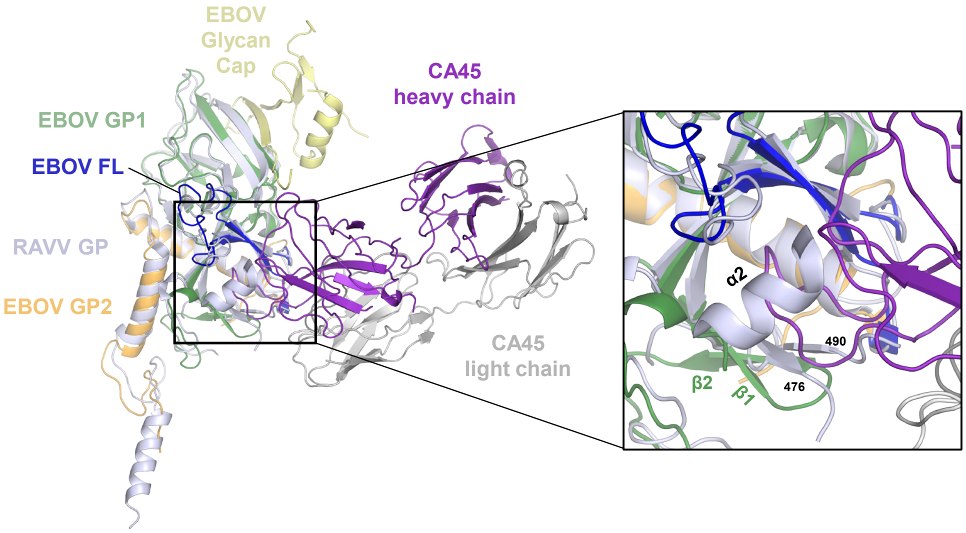

Supplementary Figure 8. Structural alignment with marburgvirus RAVV GP reveals clashes with CA45 HCDR3 loop. Shown is the structure of CA45 bound EBOV GPΔMuc superimposed with RAVV GP structure (PDB ID 6BP2, bluewhite). CA45 HCDR3 clashes with RAVV GP helix α2 and β-strand residues 474-476 and 490-492.

Supplementary Table 1 Buried surface areas on CA45 and EBOV GP*

	Buried Surface CA45 (Å^2)							
	FR1	CDR1	FR2	CDR2	FR3	CDR3	FR4	Subtotal
**CA45 HC**	108.5	114.9		5.5	57.8	614.1		900.9
**CA45 LC**		6.8	81.5	116.5				204.8
**Total**								1105.7

	Buried Surface GP (Å^2)		
	GP1	GP2 (FL)	Total
**EBOV GP**	350.0	755.0 (394.9)	1104.9

*All buried surfaces were determined with CCP4i qtPISA[1,2].

Supplementary Table 2 Sequence variability of CA45 epitope residue positions across ebolaviruses*

			EBOV n=1370 (n=210)		SUDV n=20 (n=11)		BDBV n=7 (n=3)		REST n=22 (n=9)		TAFV n=3 (n=3)	
GP Res. #	*H* (n=236)	BSA (Å^2)	aa	%Sequences	aa	%Sequences	aa	%Sequences	aa	%Sequences	aa	%Sequences
211	0.04	7.0	S	100	S	100	S	100	S	95.5 (88.9)	S	100
									G	4.5 (11.1)		
213	0.04	42.8	Y	99.93 (99.52)	Y	100	Y	100	Y	100	Y	100
			H	0.07 (0.48)								
545	0.04	94.4	E	99.93 (99.52)	E	100	E	100	E	100	E	100
			D	0.07 (0.48)								
516	0.10	11.6	H	100	H	100	H	100	H	63.6 (66.7)	H	100
									Y	36.4 (33.3)		
549	0.10	130.3	H	100	H	100	H	100	H	100	E	100
41	0.17	15.7	S	100	S	100	N	100	S	100	N	100
514	0.24	23.7	N	100	N	100	N	100	D	100	N	100
544	0.37	33.9	T	97.4 (97.7)	T	100	T	100	I	100	T	100
			I	2.6 (3.3)								
547	0.42	70.6	L	100	L	100	I	100	V	100	I	100
187	0.51	47.8	P	100	A	100	P	100	S	100	P	100
38	0.51	15.7	I	100	V	100	V	100	V	100	V	100
64	0.51	37.1	R	100	K	100	K	100	K	100	K	100
552	0.54	29.6	D	99.56 (99.05)	N	100	N	100	N	100	N	100
			N	0.44 (0.95)								
212	0.60	47.3	G	99.78 (99.05)	G	100	Y	100	Y	95.5 (88.9)	Y	100
			D	0.22 (0.95)					H	4.5 (11.1)		

*Shown is sequence variation at CA45 non-zero sequence entropy epitope positions across a dataset of 1422 available ebolavirus GP sequences, with values in parentheses corresponding to the 236 unique sequences within this dataset[3]. n, number of sequences; BSA, buried surface area; aa, amino acid; *H*, sequence entropy; Res., residue.

Supplementary Table 3 Regression analysis for shared epitope residues with CA45

	CA45 vs.:					
	**c4G7**	**KZ52**	**16F6**	**c2G4**	**IBP**	**TOR**
**PDB ID**	5KEN	3CSY	3S88	5KEL	5JQB	5JQ7
**Chain IDs**	K,M,N,O	I,J,A,B	I,J,H,L	A,B,L,H	A,B	A,B
**n**	13	9	5	6	12	15
**Equation**	Y = 0.2063*X + 1.17	Y = 0.5825*X + 0.5162	Y = 0.05219*X + 1.243	Y = -0.1019*X + 1.774	Y = 0.5661*X + 0.5464	Y = 0.6152*X + 0.531
**95% C.I. Slope**	-0.2852 to 0.6977	-0.3777 to 1.543	-0.8216 to 0.926	-0.305 to 0.1011	0.2252 to 0.907	0.3994 to 0.8311
**R^2**	0.072	0.2272	0.0119	0.3269	0.5778	0.7447
**F**	0.8535	2.058	0.03613	1.943	13.69	37.92
**DFn, DFd**	1, 11	1, 7	1, 3	1, 4	1, 10	1, 13
**P value**	0.3754	0.1946	0.8614	0.2358	0.0041	<0.0001
**Slope deviation from zero?**	Not Significant	Not Significant	Not Significant	Not Significant	Significant	Significant

n, sample size corresponding to number of shared epitope residues with CA45. C.I., confidence interval; R^2 , coefficient of determination; DFn and DFd, degrees of freedom in the numerator and denominator. All calculations performed in Graphpad Prism.

Supplementary References

1 Winn, M. D. *et al.* Overview of the CCP4 suite and current developments. *Acta Crystallogr D Biol Crystallogr* **67**, 235-242, doi:10.1107/S0907444910045749 (2011).

2 Krissinel, E. & Henrick, K. Inference of macromolecular assemblies from crystalline state. *Journal of molecular biology* **372**, 774-797, doi:10.1016/j.jmb.2007.05.022 (2007).

3 Hatcher, E. L. *et al.* Virus Variation Resource - improved response to emergent viral outbreaks. *Nucleic Acids Res* **45**, D482-D490, doi:10.1093/nar/gkw1065 (2017).
